# Reproducibility of Heart Rate Variability Indices in Children with Cystic Fibrosis

**DOI:** 10.1371/journal.pone.0151464

**Published:** 2016-03-11

**Authors:** Melitta A. McNarry, Kelly A. Mackintosh

**Affiliations:** College of Engineering, Swansea University, SA2 8PP, Wales, United Kingdom; Inserm U995-Université de Lille, FRANCE

## Abstract

Fundamental to the potential utilisation of heart rate variability (HRV) indices as a prognostic tool is the reproducibility of these measures. The purpose of the present study was therefore to investigate the reproducibility of 24-hour derived HRV indices in a clinical paediatric population. Eighteen children (10 boys; 12.4 ± 2.8 years) with mild to moderate Cystic Fibrosis (CF; FVC: 83 ± 12% predicted; FEV_1_: 80 ± 9% predicted) and eighteen age- and sex-matched controls (10 boys; 12.5 ± 2.7 years) wore a combined ECG and accelerometer for two consecutive days. Standard time and frequency domain indices of HRV were subsequently derived. Reproducibility was assessed by Bland-Altman plots, 95% limits of agreement and intra-class correlation coefficients (ICC). In both groups, there was no systematic difference between days, with the variables demonstrating a symmetrical, homoscedastic distribution around the zero line. The time domain parameters demonstrated a good to excellent reproducibility irrespective of the population considered (ICC: 0.56 to 0.86). In contrast, whilst the frequency domain parameters similarly showed excellent reproducibility in the healthy children (ICC: 0.70 to 0.96), the majority of the frequency domain parameters illustrated a poor to moderate reproducibility in those with CF (ICC: 0.22 to 0.43). The exceptions to this trend were the normalised LF and HF components which were associated with a good to excellent reproducibility. These findings thereby support the utilisation of time and relative frequency domain HRV indices as a prognostic tool in children with CF. Furthermore, the present results highlight the excellent reproducibility of HRV in healthy children, indicating that this may be a useful tool to assess intervention effectiveness in this population.

## Introduction

Cystic fibrosis (CF) is a complex genetic disease, affecting multiple organs through the disruption of the CF transmembrane conductance regulator protein. In addition to its clinical presentation, there is a growing body of evidence of concomitant autonomic neuropathy in CF. Specifically, an increased sensitivity to α-adrenergic stimulation of pupil dilatation and increased responsiveness to cholinergic stimulation of pupil constriction have been shown [1], as well as a reduced cardiovascular sensitivity to β-adrenergic stimulation [2].

Heart rate variability (HRV) provides a valuable, non-invasive insight into the (dys)function of the autonomic nervous system [3, 4] and is an established indicator of an increased risk of cardiac mortality [4–6]. Furthermore, HRV is highly sensitive, with suggestions that negative changes in HRV may predate clinical symptoms of autonomic neuropathy by several years [7], thereby potentially offering a useful prognostic tool. Recently, abnormal HRV has been reported in both children [8] and adults [9] with CF, providing further evidence of autonomic neuropathy in this population. However, it is pertinent to note the directionality of these abnormalities were in direct contradiction; Florencio et al. [8] reported an increased sympathetic tone whilst Szollosi et al. [9] reported a decreased sympathetic tone. Furthermore, a significant correlation was present between HRV and disease severity in the adult study only. Whilst these discrepancies may be largely attributable to the pathological progression of the disease and the desensitisation of the β-adrenergic receptors consequent to chronic β-adrenergic agonist use, methodological explanations cannot be precluded, such as differences in the conditions under which HRV was derived (pre/post exercise vs. supine rest). Moreover, fundamental to the interpretation of such between-group differences in HRV, and thus the their clinical significance, is verification of the reliability and reproducibility of these variables in patients with CF. A substantial body of evidence is available regarding the reliability of HRV parameters during 24-hour recordings (e.g. [10, 11–13]), short-term (e.g. 2–15 min) recordings under stationary, stable conditions (e.g. [14, 15–17]) and during exercise [18]. However, considerably less is known regarding the reproducibility of such measures in children; the limited data available suggests HRV to be unreliable when derived from short-term recordings in healthy children [19, 20]. Despite the potential clinical significance, the applicability of such findings to, and thus the reproducibility of HRV in, children with CF is presently unclear.

Therefore, the purpose of the present study was to investigate inter- and intra-participant reproducibility of time and frequency domain HRV derived from 24-hour recordings.

## Methods

### Study Population and Anthropometry

Following ethical approval from the Bromley NHS research ethics committee (REC reference: 13/LO/1907), eighteen patients (8 girls; Table 1) with mild-to-moderate CF were recruited from an outpatient CF clinic in South Wales (United Kingdom). Cystic Fibrosis was confirmed by a sweat chloride > 60 mmol·L^−1^/100 mg and genotyping (8 ΔF508 Homozygote, 10 ΔF508 Heterzygote; 4 CF-related liver disease.). Stable lung function within 10% of best in the preceding 6 months and no increase in symptoms or weight loss 2 weeks prior to testing were obligatory. Unstable non-pulmonary comorbidities or acute infections warranted exclusion. Disease severity was graded using routine clinical measurements obtained as part of patients' annual review by their multidisciplinary CF clinical care team (Table 1). Eighteen age- and sex-matched, apparently healthy children were recruited from local primary and secondary schools to act as a comparison group. Written informed consent and assent were obtained from parents/guardians and patients, respectively. All patients were instructed to continue maintenance medications as usual throughout the duration of their study involvement.

**Table 1:** Participant characteristics.

	Total	Cystic Fibrosis	Controls
***n***	36	18	18
**Age (yrs)**	12.6 ± 2.7	12.4 ± 2.8	12.5 ± 2.7
**Stature (m)**	1.48 ± 0.14	1.46 ± 0.14	1.51 ± 0.13
**Mass (kg)**	44.24 ± 12.99	41.16 ± 12.51	47.52 ± 13.04
**Waist circumference (m)**	0.67 ± 0.08	0.66 ± 0.07	0.67 ± 0.09
**BMI (kg·m**^**2**^**)**	19.6 ± 3.4	18.8 ± 2.8	20.5 ± 3.8
**Maturity offset (yrs from PHV)**	-1.28 ± 3.00	-1.04 ± 2.42	-1.54 ± 3.57
**FVC (% predicted)**	84 ± 15	83 ± 12	85 ± 18
**FEV**_**1**_ **(% predicted)**	85 ± 14	80 ± 9	89 ± 17
**Daily MVPA (mins)**	98 ± 57	118 ± 53	77 ± 54^*^

Mean ± S.D. PHV, peak height velocity; FVC, forced vital capacity; FEV_1_, forced expiratory volume in 1 second; MVPA, moderate-to-vigorous physical activity.

^*^ significant difference between control and Cystic fibrosis

Body mass (Seca 220; Hamburg, Germany), stature and sitting stature (Seca 220; Hamburg, Germany) were measured to the nearest 0.01 kg and 0.01 m, respectively. Waist and hip circumference were measured to the nearest 0.01 m using a non-elastic anthropometric tape (Seca Ltd., Birmingham, UK) at the narrowest point between the bottom of the ribs and the iliac crest and widest point around the hips, respectively. Forced vital capacity (FVC) and forced expiratory volume in 1-s (FEV1) were also assessed using flow-volume loop spirometry (MicroMedical MicroLoop 3535, Numed, Sheffield, UK). The best of three consistent exhalations (<5% variability) was recorded. All lung function measurements were expressed as a percentage predicted normal, using appropriate reference data [21].

### Protocol

At their routine visits to the clinic, participants were provided with an Actiheart monitor to measure inter-beat interval and a tri-axial accelerometer to assess habitual physical activity levels. Participants were asked to wear both monitors for three days to enable the comparison of two complete 24 hour periods.

### Measurements

Inter-beat interval was measured using a combined heart rate and accelerometer (Actiheart, Camntech, Cambridge, UK). These are flat, lightweight (<10g) devices for which high levels of intra- and inter-instrument reliability, as well as good validity measures, have been reported [22]. Following preparation of respective skin areas using an abrasive alcohol wipe, the Actiheart was firmly fixed to an electrode placed just below the apex of the sternum (mid-way below the V1 and V2 positions) while the wire running from the monitor was fixed to an electrode placed on the same horizontal level and as lateral as possible. This placement is associated with higher ECG amplitudes and a lower level of noise from movement artefacts [22]. The analogue signal was band-pass filtered (10–35 Hz), sampled with a frequency of 128 Hz, and processed by a real time QRS-detection algorithm. The QT and RR data also underwent human visual examination in order to verify the accuracy of the data prior to subsequent analysis. When either the RR or QT interval was considered to be anomalous, both the RR and QT data points were removed from the data set. This occurred infrequently (and mainly during exercise), overall resulting in fewer than 1% of the data being removed.

To account for differences in physical activity levels between the measurement days, a tri-axial accelerometer (wGT3X; ActiGraph, LLC, Fort Walton Beach, FL) was worn by participants on the right mid-axilla line at the level of the iliac crest. Patients were instructed to wear the accelerometer, measuring at 100 Hz, for three consecutive days, only removing during water-based activities.

### Data Analysis

Heart rate variability variables were quantified in the time domain (RMSSD: square root of the mean of the sum of the squares of differences between adjacent RR intervals; SDNN: standard deviation of all RR intervals; Triangle Index (TI): total number of RR intervals divided by the number of RR intervals in the modal bin [bin width = 8ms]) and the frequency domain (Very Low Frequency (VLF) power: 0.017 to 0.04 Hz, Low frequency (LF) power: 0.04 to 0.15 Hz, high frequency (HF) power: 0.15 to 0.4 Hz, Total Power (TP): 0.017 to 0.4 Hz,), normalised HF (HFn, HF/(Total Power-VLF)) and LF (LFn, LF/(Total Power-VLF)) and the ratio of LF to HF according to the Task Force guidelines on HRV [4]. Prior to the frequency domain analysis procedures RR interval data were re-sampled using a sampling frequency of 2 Hz and then linearly de-trended and Hanning windowed in consecutive one-minute segments; the power spectral density of each segment was then calculated using the Welch periodogram method, using short-term Fourier transformation and a 50% overlap between adjacent segments.

### Calculation of physical activity levels

In the absence of universally agreed cut-points to classify children’s physical activity intensities, the cut-points of Evenson et al. [23] were selected on the basis of a methodologically rigorous comparison study, which concluded that they have acceptable accuracy across a range of intensities and ages. Non-accelerometer wear time was defined as at least 20 min periods of consecutive zero counts [24], with wear time criteria at least 540 minutes per day. These criteria have been shown to yield a reliability of 0.9 suggesting a high degree of consistency across days [25].

### Statistical Analyses

Initially, data were assessed for normality by the Shapiro-Wilks test; those revealed as skewed were log-transformed prior to subsequent analyses. Bland-Altman plots of the difference between paired measurements as a function of their mean were constructed to allow visualisation of any systematic change between the tests and to assess the data for heteroscedasticity. The absence of any systematic between-test change was further verified by paired samples t-tests.

The standard error of measurement (SEM) was estimated from the results of an ANOVA as the square root of the within-subject mean square. The 95% limits of random variation were subsequently derived from the SEM values, with the limits back-transformed for those HRV variables initially log-transformed. Intraclass correlation coefficients (ICC) were also determined from the mean square values of the ANOVA.

All analyses were conducted using the PASW Statistics package version 21 (SPSS, Chicago, IL). Statistical significance was accepted as *P*<0.05.

## Results

**Fig 1:**
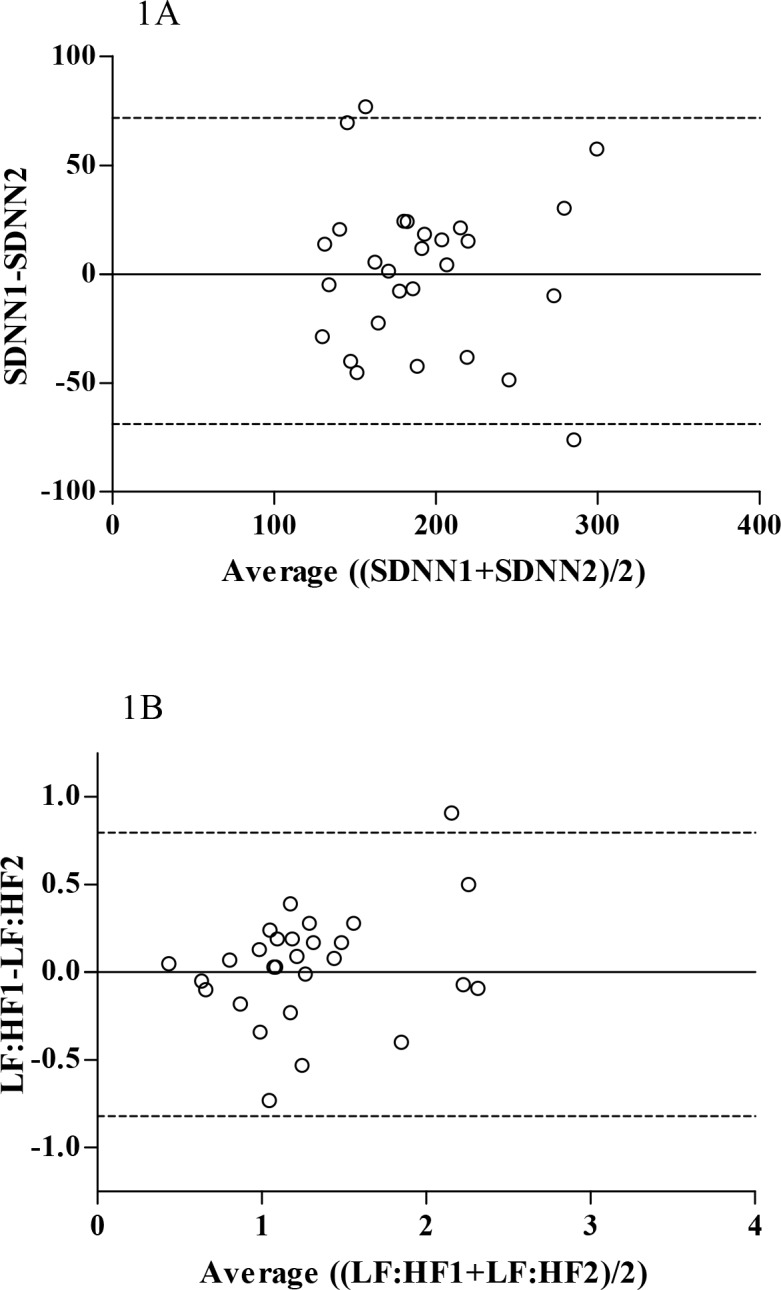
Representative Bland-Altman plots of the difference between two repeat measurements. The points were systematically distributed around the zero line indicating a homoscedastic distribution.

Descriptive statistics for the HRV and physical activity variables are shown in Table 2. The time spent in moderate-to-vigorous physical activity (MVPA) was above the national recommended guidelines of 60 minutes every day [26], with no significant between-day differences. In both children with and without CF, the time domain parameters of the HRV did not demonstrate a skewed distribution according to the Shapiro-Wilks test (*P* > 0.05), the VLF, LF and HF indices demonstrated a significantly skewed distribution (*P* < 0.05). Normalisation and log-transformation of these variables elicited a normal distribution (no skew; *P* > 0.05). In both populations and the sample population as a whole, both time domain and spectral domain parameters revealed a symmetrical distribution of points around the zero line according to Bland-Altman plots, indicating the absence of a systematic change as a function of the mean (Fig 1). Such an absence was further verified by non-significant paired samples t-tests (*P*>0.05). Furthermore, the plots revealed the data to be homoscedastic.

**Table 2:** HRV variables in children with CF and healthy age and sex-matched children.

	Total		CF		Controls	
	Mean	Mean ∆	Mean	Mean ∆	Mean	Mean ∆
**SDNN (ms)**	192 ± 52	-1.4	198 ± 52	-17.1	188 ± 52	10.3
**SDANN (ms)**	151 ± 47	-0.6	157± 54	-16.4	147 ± 43	10.2
**RMSSD (ms)**	113 ± 43	3.5	126 ± 39	-9.4	103 ± 43	13.3
**VLF (ms**^**2**^**)**	5207 ± 8806	-125	4095 ± 3176	-1446	6042 ± 11397	866
**LF (ms**^**2**^**)**	7339 ± 14100	90.0	5598 ± 4476	-1747	8645 ± 18378	1468
**LFn (nu)**	55 ± 11	0.3	51 ± 12	1.1	58 ± 9	-0.3
**HF (ms**^**2**^**)**	5086 ± 6368	1.9	4928 ± 2851	-1442	5204 ± 8141	1085
**HFn (nu)**	45 ± 11	-0.3	49 ± 12	-1.1	42 ± 9	0.3
**TP (ms**^**2**^**)**	9239 ± 10136	-1366.4	14620 ± 10071	-4635	19891 ± 37787	3419
**TI**	44 ± 13	1.8	42 ± 16	1.42	45 ± 11	2.1
**LF:HF**	1.3 ± 0.6	0.02	1.2 ± 0.5	0	1.5 ± 0.6	0.03

Mean ± S.D. RMSSD, square root of the mean of the sum of the squares of differences between adjacent RR intervals; SDNN, standard deviation of all RR intervals; SDANN, standard deviation of the average RR interval; VLF, very low frequency power; LF, low frequency power; HF, high frequency power (0.15–0.4 Hz); TP, total power; LFn, normalised low frequency power; HFn, normalised high frequency power; TI, Triangular index; mean Δ, mean differences between measures.

Reproducibility indices for the HRV parameters are shown in Table 3. The time domain parameters demonstrated a good to excellent reproducibility irrespective of the population considered. In contrast, whilst the frequency domain parameters similarly showed excellent reproducibility in the healthy children, the majority of the frequency domain parameters illustrated a poor to moderate reproducibility in those with CF. The exceptions to this trend were the normalised LF and HF components which were associated with a good to excellent reproducibility.

**Table 3:** Reliability indices of HRV parameters.

	Total			CF			Controls		
	SEM	95% CI (lower, upper)	ICC	SEM	95% CI (lower, upper)	ICC	SEM	95% CI (lower, upper)	ICC
**SDNN (ms)**	0.49	0.39, 0.67	0.77	0.42	0.29, 0.71	0.86	0.49	0.36, 0.76	0.78
**SDANN (ms)**	0.67	0.53,0.92	0.56	0.70	0.49, 1.23	0.56	0.60	0.44, 0.92	0.67
**RMSSD (ms)**	0.51	0.41, 0.70	0.75	0.55	0.39, 0.94	0.73	0.47	0.34, 0.72	0.81
**TP (ms**^**2**^**)**	0.62	0.49, 0.84	0.63	0.89	0.63, 1.51	0.24	0.22	0.17, 0.35	0.96
**VLF (ms**^**2**^**)**	0.32	0.25, 0.43	0.91	0.90	0.64, 1.52	0.22	0.23	0.17, 0.36	0.95
**Ln VLF (ln ms**^**2**^**)**	0.57	0.45, 0.78	0.69	0.78	0.55, 1.32	0.43	0.44	0.33, 0.69	0.83
**LF (ms**^**2**^**)**	0.28	0.22, 0.38	0.93	0.87	0.62, 1.48	0.26	0.21	0.16, 0.32	0.96
**Ln LF (ln ms**^**2**^**)**	0.61	0.48, 0.82	0.65	0.80	0.56, 1.35	0.40	0.51	0.38, 0.79	0.77
**LFn (nu)**	0.41	0.33, 0.56	0.84	0.45	0.32, 0.76	0.83	0.42	0.31, 0.65	0.85
**HF (ms**^**2**^**)**	0.38	0.30, 0.51	0.87	0.80	0.57, 1.35	0.40	0.28	0.21, 0.43	0.93
**Ln HF (ln ms**^**2**^**)**	0.55	0.44, 0.75	0.71	0.79	0.56, 1.33	0.42	0.47	0.35, 0.73	0.80
**HFn (nu)**	0.41	0.33, 0.56	0.84	0.45	0.32, 0.76	0.83	0.42	0.31, 0.65	0.85
**TI**	0.41	0.32, 0.56	0.84	0.27	0.19, 0.46	0.94	0.57	0.42, 0.89	0.70
**LF:HF**	0.48	0.38, 0.65	0.78	0.53	0.38, 0.90	0.76	0.48	0.35, 0.74	0.79

SEM, standard error of measurement; CI, confidence interval; ICC, intraclass correlation coefficient; SDNN, standard deviation of all RR intervals; SDANN, standard deviation of the average RR interval; RMSSD, square root of the mean of the sum of the squares of differences between adjacent RR intervals; VLF, very low frequency power; LF, low frequency power; HF, high frequency power (0.15–0.4 Hz); TP, total power; LFn, normalised low frequency power; HFn, normalised high frequency power; Ln, natural log; TI, Triangular index.

## Discussion

The primary purpose of the present study was to determine the reproducibility of HRV indices derived from consecutive 24 hour measurement periods in children with CF compared to age- and sex-matched controls. The main findings are that both time and frequency domain parameters demonstrate a high level of reproducibility, although this reproducibility appears to be slightly lower in certain parameters in children with CF. Specifically, time domain indices demonstrate high reproducibility whilst frequency domain parameters were more variable. The exception to the latter is the normalised high and low frequency components, which evidenced a comparable reproducibility to the time domain measures. These findings therefore support the utilisation of 24 hour derived time domain HRV indices to monitor and assess autonomic function in both children with and without CF.

The clinical utility of HRV, which provides a non-invasive insight into autonomic (dys)function is well evidenced; negative changes in HRV have been suggested to predate clinical manifestations of autonomic neuropathy by several years. Fundamental to this potential application of HRV as a prognostic tool is the reliability and reproducibility of the measurements over time. However, despite the importance of this, the present study is the first to investigate the reproducibility of HRV in a clinical, paediatric population. Although comparisons to studies in healthy children must be made with caution due to differences in the study populations and methodologies, such studies have reported a questionable reliability during short-term recordings. Specifically, at rest, Winsley [19] reported time and frequency domain indices to demonstrate ICC ranging from 0.14 to 0.49. More recently, Leicht and Allen [20] found considerable higher ICC for frequency domain measures at rest, however these were associated with large coefficient of variations and wide ratio limits of agreement. The present findings in healthy children therefore largely contradict these earlier studies, demonstrating a high level of reproducibility of both time and frequency domain measures during habitual activities. This discrepancy may be related to the longer measurement period in the present study, minimising the influence of short-term fluctuations, or to different measurement frequencies. It is pertinent to note that, whilst lower than observed in the current control children, the reproducibility presently observed in the children with CF is largely in agreement with the earlier studies, finding ICC ranging from 0.56 to 0.86 and 0.22 to 0.83 for time and frequency domains, respectively. Furthermore, the greater reproducibility associated with the time domain measures are in accord with previous studies and, indeed, our hypothesis. This greater reproducibility is likely, at least in part, to be attributable to these parameters being less influenced by non-stationarities in the data [27–29]. Given this, and the superior interpretability associated with time domain measures derived from 24 hour measurement periods [4], preferential reliance on these parameters should perhaps be advocated. In agreement with findings in both healthy children [19, 20] and adults [17, 30], a greater reproducibility was presently associated with relative measures of HF and LF compared to their absolute counterparts. This procedure tends to minimise the effect of changes in total power on the LF and HF components [4]; given the positive relationship between the total variance of HRV with the length of the analysed recording [4], it would therefore be expected that this would be associated with a better reliability.

The lower reproducibility observed in those with CF compared to their age- and sex-matched controls is in accord with previous studies in adults comparing the reproducibility of HRV parameters in healthy and patient groups [30]. Whilst the basis for this decreased reproducibility remains to be elucidated, it may be postulated to be related to daily variation in factors inherent to the disease itself as there were no differences in mean moderate-to-vigorous physical activity levels between days within or between groups. Further work is required to determine if this reduced reproducibility is associated with early indicators of the onset of autonomic dysfunction.

The derivation of the present results from 24 hour measurements requires certain factors to be considered when interpreting the findings. Specifically, the highest reliability and reproducibility of HRV variables have previously been reported when external factors, such as time of recording, overnight fast and the restriction of tobacco, caffeine and exercise, are as stringently enforced. Indeed, differences in the control of these conditions is likely to underlie the higher reliability reported by Leicht and Allen [20] relative to Winsley [19]. Nonetheless, these conditions could not be controlled in this, more ecologically valid, investigation of HRV reproducibility and thus one might anticipate a lower reproducibility to be manifest in this study. Conversely, the present study found that this was not the case. Such findings may be attributable to the similar physical activity levels observed in participants on the ECG measurement days. Furthermore, since it has been suggested HRV reliability may be related to the absolute level of HRV, with a lower HRV associated with a greater reliability, the present findings may also be a reflection of a lower HRV in this patient group relative to healthy children. However, the applicability of this explanation is presently unclear as it remains to be elucidated as to whether global HRV is depressed in CF as has been reported in other pathological conditions, such as heart failure (e.g. [31, 32]), hypertension [33] and diabetes (e.g. [34, 35]).

Although not the purpose of the present investigation, it is pertinent to note the similarity of the present levels of HRV to those previously reported in children with CF by Florencio et al [8]. However, both of these studies, reporting an elevated sympathetic tone, are in contradiction to recent findings in adults with CF which showed a lower sympathetic tone. These findings, which suggest the presence of a concomitant autonomic neuropathy in those with CF, may indicate an age-related modulation of HRV which warrants further investigation. Alternatively, or in addition, these child-adult differences may be associated with the progression of this disease and could reflect the desensitisation of the β-adrenergic receptors consequent to chronic β-adrenergic agonist use.

Abnormal HRV has recently been reported in CF, suggesting a concomitant autonomic neuropathy. However, the manifestation of this abnormality has been suggested to be modulated with age. Specifically, in accord with the present findings, Florencio et al. [8] showed a higher sympathetic tone in children whereas Szollosi et al. [9] reported a lower sympathetic tone in adults with CF. The similarity of the current 24 hour derived values to those of Florencio et al. [8] from a 20 min supine rest is noteworthy. These discrepant findings in children and adults may be indicative of the progression of this disease and could reflect the desensitisation of the β-adrenergic receptors consequent to chronic β-adrenergic agonist use.

Due to the nature of this study, there are several methodological aspects that should be considered. Firstly, although higher than previous studies in healthy children investigating the reliability of HRV measures [19, 20], the present sample size was limited. However, given the prevalence of CF and thus the overall population available from which to recruit participants, we believe that the present results provide relevant and generalizable conclusions. Moreover, the inclusion of age and sex-matched control significantly strengthens the present analyses. Perhaps more important with regard to the current sample is the age range of the participants given that HRV is suggested to be modulated by age. Future studies should seek to recruit evenly across the maturational and development stages to account for this potential effect.

In conclusion, this study shows for the first time in a clinical paediatric population that time and relative frequency domain parameters derived from 24 hour measurements demonstrate good-to-excellent reproducibility. These findings thereby support the utilisation of HRV indices as a prognostic tool in children with CF. Furthermore, the present results highlight the excellent reproducibility of HRV in healthy children, indicating that this may be a useful tool to assess intervention effectiveness in this population.
